# Rational design of non-toxic GOx-based biocatalytic nanoreactor for multimodal synergistic therapy and tumor metastasis suppression

**DOI:** 10.7150/thno.65399

**Published:** 2021-10-25

**Authors:** Lifeng Hang, Tao Zhang, Hua Wen, Meng Li, Lianbao Liang, Xinfeng Tang, Chunze Zhou, JunZhang Tian, Xiaofen Ma, Guihua Jiang

**Affiliations:** 1 The Department of Medical Imaging, Guangdong Second Provincial General Hospital, Guangzhou, 518037, P. R. China.; 2 Key Laboratory of Materials Physics, Institute of Solid State Physics, HFIPS, Chinese Academy of Sciences, Hefei, 230031, P. R. China.; 3 Division of Molecular Medicine, Hefei National Laboratory for Physical Sciences at Microscale, School of Life Sciences, University of Science and Technology of China, Hefei, 230001, P. R. China.; 4 Interventional Radiology Department, The First Affiliated Hospital of USTC, Division of Life Sciences and Medicine, University of Science and Technology of China, Hefei, 230001, P. R. China.

**Keywords:** Glucose oxidase, tirapazamine, metal-organic framework, biocatalytic nanoreactor, starvation therapy

## Abstract

**Rationale:** Glucose oxidase (GOx)-based biocatalytic nanoreactors can cut off the energy supply of tumors for starvation therapy and deoxygenation-activated chemotherapy. However, these nanoreactors, including mesoporous silica, calcium phosphate, metal-organic framework, or polymer nanocarriers, cannot completely block the reaction of GOx with glucose in the blood, inducing systemic toxicity from hydrogen peroxide (H_2_O_2_) and anoxia. The low enzyme loading capacity can reduce systemic toxicity but limits its therapeutic effect. Here, we describe a real 'ON/OFF' intelligent nanoreactor with a core-shell structure (GOx + tirazapamine (TPZ))/ZIF-8@ZIF-8 modified with the red cell membrane (GTZ@Z-RBM) for cargo delivery.

**Methods:** GTZ@Z-RBM nanoparticles (NPs) were prepared by the co-precipitation and epitaxial growth process under mild conditions. The core-shell structure loaded with GOx and TPZ was characterized for hydrate particle size and surface charge. The GTZ@Z-RBM NPs morphology, drug, and GOx loading/releasing abilities, system toxicity, multimodal synergistic therapy, and tumor metastasis suppression were investigated. The *in vitro* and *in vivo* outcomes of GTZ@Z-RBM NPs were assessed in 4T1 breast cancer cells.

**Results:** GTZ@Z-RBM NPs could spatially isolate the enzyme from glucose in a physiological environment, reducing systemic toxicity. The fabricated nanoreactor with high enzyme loading capacity and good biocompatibility could deliver GOx and TPZ to the tumors, thereby exhausting glucose, generating H_2_O_2_, and aggravating hypoxic microenvironment for starvation therapy, DNA damage, and deoxygenation-activated chemotherapy. Significantly, the synergistic therapy effectively suppressed the breast cancer metastasis in mice and prolonged life without systemic toxicity. The *in vitro* and *in vivo* results provided evidence that our biomimetic nanoreactor had a powerful synergistic cascade effect in treating breast cancer.

**Conclusion:** GTZ@Z-RBM NPs can be used as an 'ON/OFF' intelligent nanoreactor to deliver GOx and TPZ for multimodal synergistic therapy and tumor metastasis suppression.

## Introduction

Glucose oxidase (GOx), an endogenous oxidoreductase, has attracted much attention for tumor starvation and multimodal synergistic therapy due to its ability to consume glucose of cancer cells and modulate the cancer hypoxic microenvironment 1-6. GOx delivery strategies are currently based on porous materials, including organic microparticles, mesoporous silica, MnCaP, and metal-organic frameworks (MOFs) 7-15. However, these nanocarriers, directly immobilizing enzymes through surface attachment, pore encapsulation, covalent linkage, or co-precipitation, have disadvantages, such as low enzyme loading rate, enzyme leaching, the ineffective release of enzymes, and toxic hydrogen peroxide (H_2_O_2_) production in the blood, severely limiting their practical application for GOx delivery 16-20. Therefore, the rational structural design of GOx nanocarriers is still challenging.

Although GOx can cut off the energy supply of tumors, GOx-based nanoreactors generate H_2_O_2_ and consume oxygen (O_2_) during circulation, inducing adverse effects in the body. Zhang and colleagues reported a cascade bioreactor mem@catalase@GOx@PCN-224 that could consume glucose and decompose endogenous H_2_O_2_ to reduce the O_2_ consumption and minimize systemic toxicity 21. Also, the reported nano-clustered cascaded enzymes BCE@(GOx+CAT) prepared by self-assembly *via* PEG-b-PHEMA_CMA_ could reduce systemic toxicity 22. However, the low enzyme loading capacity and long production cycle hinder the use of the nanoplatforms as GOx nanoreactors. Another study by Shi et al. employed mesoporous silica nanoparticles (NPs) as GOx carriers based on electrostatic interactions 23, 24. Wu and co-workers group reported a self-amplified nanoreactor (HSA-GOx-TPZ-Fe^3+^-TA) with sustainable and cascade anticancer performance 25. These strategies may suffer from enzyme leaching and low loading capacity.

As a type of porous material, MOFs have recently been used as enzyme delivery systems due to their high surface area, tunable pore size, and ultrahigh porosity 26-32. In particular, Zeolitic imidazolate framework-8 (ZIF-8) could efficiently encapsulate enzymes under mild conditions and afford unprecedented protection from chemical, thermal, and biological degradation while maintaining of bioactivity 33, 34. The encapsulated enzymes could be released under an acid physiological environment 35. Therefore, ZIF-8 represents a thermally and chemically stable excellent candidate for enzymes delivery. Qu group encapsulated GOx and prodrug tirapazamine (TPZ) in ZIF-8 wrapped with an erythrocyte membrane for starvation-activated cancer therapy 36. However, Itamar and co-workers demonstrated that integration of GOx in ZIF-8 enhanced its catalytic activity as a nanoreactor in the glucose solution under neutral conditions (pH = 7.4) 37, 38, producing toxic H_2_O_2_ during circulation in the body and inducing severe systemic adverse effects. Therefore, a rational structural design of GOx/ZIF-8 nanoreactor as an intelligent therapeutic system for implementing real 'ON/OFF' is required to minimize systemic toxicity.

The epitaxial growth process has been successfully employed to fabricate MOF shells on the surface of MOF seeds *via* heterogeneous nucleation 39, 40. The thickness of the shell could be easily controlled by varying the molar feeding ratio. Coating a ZIF-8 shell on the GOx/ZIF-8 nanoreactor, forming a core-shell structure, would separate the glucose and GOx spatially in the neutral physiological environment. In the acidic tumor microenvironment, the ZIF-8 nanoreactor would decompose under acidic conditions, and the released GOx would consume the O_2_ and intratumoral glucose during the GOx-based starvation therapy. Furthermore, consumption of intratumoral O_2_ would aggravate the hypoxic microenvironment, activating the hypoxic prodrug inducing cell apoptosis 41, 42. During GOx-based starvation therapy, the generated H_2_O_2_ as reactive oxygen species (ROS) can cause cell apoptosis by damaging cellular DNA. Therefore, the core-shell structure nanoreactor, encapsulating GOx and hypoxic prodrug in the core, can on-demand release of the enzyme and hypoxia drugs under acidic conditions, reduce systemic toxicity, and realize effective multimodal cancer therapy.

**Figure 1:**
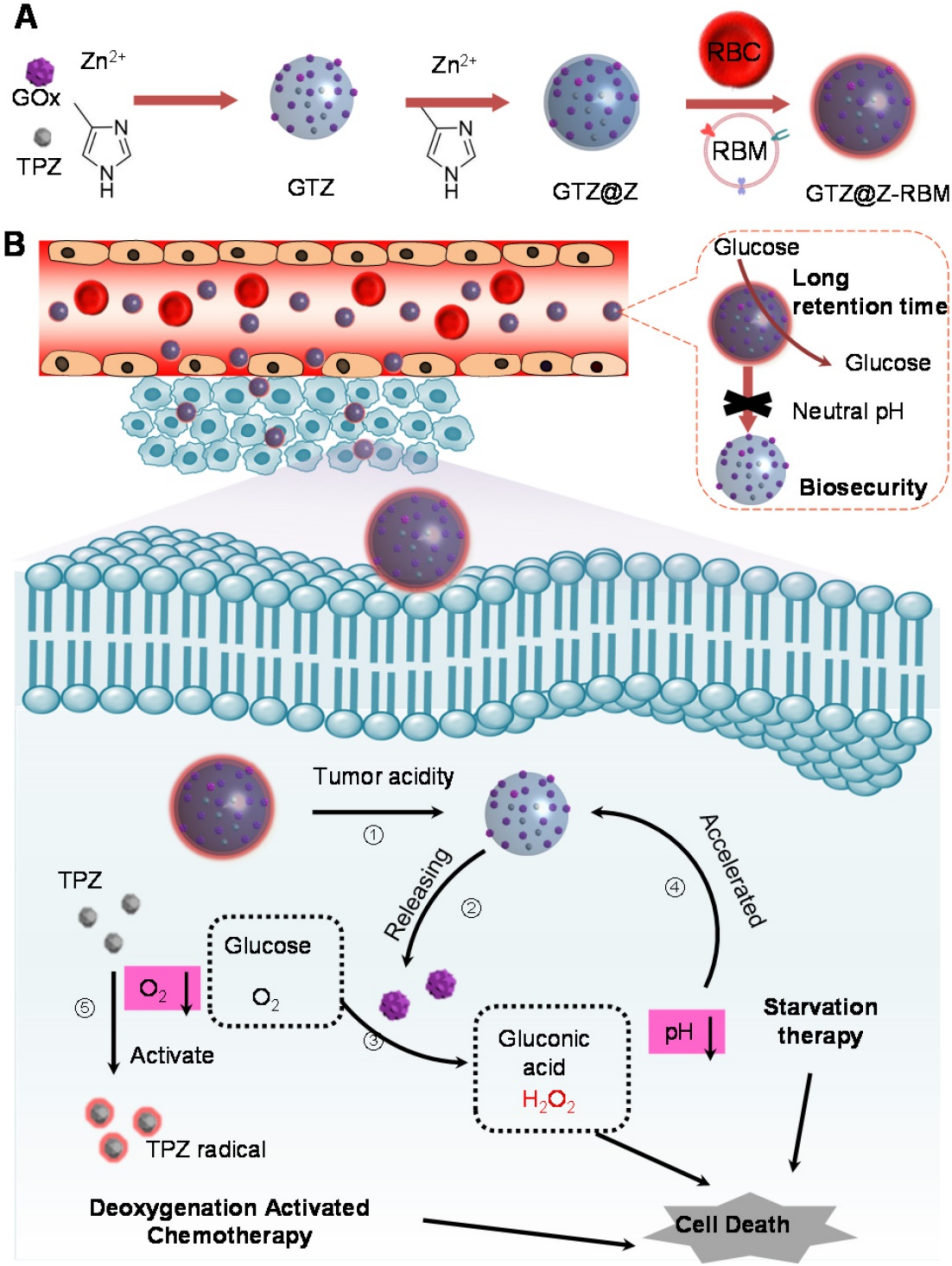
**Schematic illustration** of (**A**) GTZ@Z-RBM nanoreactor synthesis by co-precipitation and epitaxial growth processes, and (**B**) The 'ON/OFF' GOx releasing behavior from GTZ@Z-RBM nanoreactor with long retention time, biosecurity, and multimodal cancer therapy.

Herein, we developed a core-shell structure nanoreactor as an 'ON/OFF' intelligent therapeutic system, encapsulating GOx and TPZ for multimodal cancer therapy. As illustrated in **Figure 1A**, TPZ and GOx were encapsulated in the nanoscale ZIF-8 (defined as GTZ) by the co-precipitation strategy under mild condition. Subsequently, another ZIF-8 shell was fabricated on the surface of GTZ (defined as GTZ@Z) by epitaxial growth. The nanoreactors were further coated with the red cell membrane (RBM) to obtain GTZ@Z-RBM, improving the biocompatibility with prolonged blood circulation characteristics. In **Figure 1B**, the stability of the ZIF-8 shell under physiological conditions can prevent premature exposure of GOx to blood glucose, but the GTZ@Z-RBM selectively releases of GOx and TPZ under the tumor acid microenvironment. The synthesized GTZ@Z-RBM NPs as 'ON/OFF' nanoreactors could reduce system toxicity and release GOx on-demand under an acid condition. Moreover, the GTZ@Z-RBM can be used as a multimodal therapeutic system for synergistic cancer therapy. The released GOx can efficiently decompose glucose to starve tumor cells, and the produced gluconic acid can reduce pH to accelerate the decomposition of the nanoreactor. Then, during the starvation therapy, the aggravated hypoxic microenvironment can transform the prodrug TPZ into a highly cytotoxic radical for hypoxia-activated chemotherapy to induce cell apoptosis. The high concentration of generated H_2_O_2_ as a ROS species can also cause cell apoptosis. The GTZ@Z-RBM nanoreactor can suppress tumor metastasis and prolong the survival of mice with metastatic breast cancer. Taken together, GTZ@Z-RBM nanoreactor can minimize systemic toxicity and realize effective multimodal cancer therapy, and simultaneously suppress tumor metastasis.

## Methods

### Materials

Glucose oxidase (GOx, 200 u/mg), Tirapazamine (TPZ), 3-(4,5-dimethylthiazol-2-yl)-2,5-diphenyl-tetrazolium bromide (MTT) and 4,6-diamidino-2-phenylindole (DAPI) were purchased from Sigma-Aldrich. Zinc nitrate (Zn(NO_3_)_2_ 6H_2_O), catalase (100 u/mg), H_2_O_2_, 2-methylimidazole, fluorescein isothiocyanate (FITC), 3,3,3',3'-tetramethylindodicarbocyanine perchlorate (DiD), glucose, titanium oxysulfate (TiOSO_4_·XH_2_SO_4_·8H_2_O), sulfuric acid (H_2_SO_4_), and acetone were acquired from Sinopharm Chemical Reagent co. LTD. Fetal bovine serum (FBS) and Dulbecco's modified eagle medium (DMEM) with low glucose (1 g L^-1^) were bought from Gibco BRL (Eggenstein, Germany). Milli-Q water (18.2 MΩ·cm) was obtained from the Milli-Q System (Millipore, Bedford, MA, USA).

### Synthesis of GTZ@Z-RBM nanoreactor

Firstly, GOx and TPZ (4 mg each) were added to 1 mL deionized water, followed by 6.6 mg of Zn(NO_3_)_2_ 6H_2_O. Subsequently, 360 mg of 2-methylimidazole was added to the solution. The mixture turned milky almost instantly after mixing. After stirring for 30 min, the product (GTZ) was collected by centrifugation at 6000 rpm for 10 min, washed with deionized water three times, and re-dispersed in deionized water.

Next, GTZ@Z core-shell NPs were prepared using the epitaxial growth method by adding 36.4 mg of 2-methylimidazole and 1 mg of Zn(NO_3_)_2_ 6H_2_O to 1 mL of GTZ suspension liquid. Subsequently, the mixture ages at room temperature for 30 min. The product (GTZ@Z) was collected by centrifugation at 6000 rpm for 10 min, washed with deionized water three times, and re-dispersed in deionized water.

Finally, GTZ@Z-RBM nanoreactors were prepared by coating NPs with RBM. For this, RBM (1 mg) and GTZ@Z NPs (1 mg mL^-1^) were mixed via sonication for 10 min.

### *In vitro* anticancer studies

4T1 breast cancer cells were seeded in 96-well plates at a density of 3000 cells well^-1^ (100 µL total volume well^-1^) and allowed to grow for 24 h, and then incubated in DMEM (pH = 7.4 or 6.0) with various concentrations PBS, ZIF-8, TPZ, GTZ-RBM, GZ@Z-RBM, or GTZ@Z-RBM at 37 °C, in a humidified chamber containing 1% O_2_ and 5% CO_2_ for 24 h. To evaluate cell cytotoxicity, MTT solution (10 µL) was added to each microtiter plate well and incubated for an additional 4 h. The MTT containing culture medium was then replaced with 100 µL of DMSO. Absorbance values of MTT-formazan were determined with the Bio-Rad model-680 microplate reader at 490 nm.

### Anti-tumor efficacy *in vivo*

The 4T1 tumor-bearing mice were randomly divided into five groups (n = 5 per group) and PBS, ZIF-8-RBM, TZ@Z-RBM, GZ@Z-RBM, GTZ@Z-RBM with dose of 200 U/kg GOx, and 2 mg/kg TPZ were intravenously (*i.v.*) injected twice a week. The tumor growth was monitored every three days by measuring the perpendicular diameter of tumors (*i.e.;* length and width, respectively) using calipers. The volume was calculated according to the formula: Tumor volume (mm^3^) = 0.5 × length × width^2^. The weight of the mice was also determined every three days.

### Inhibition of tumor metastasis

4T1-luc cells (5 × 10^4^) were administered by *i.v.* injection into mice. The mice with metastatic tumors were randomly divided into five groups (n = 5), and treated with PBS, ZIF-8-RBM, TZ@Z-RBM, GZ@Z-RBM, and GTZ@Z-RBM. Tumor burden was monitored by IVIS imaging on days 7, 15, and 25 and quantitatively expressed as luminescence signal intensity in the region of interest (ROI). The death of the mice was also monitored and recorded daily.

## Results and Discussion

### Characterization of GTZ@Z-RBM nanoreactor

**Figure 2:**
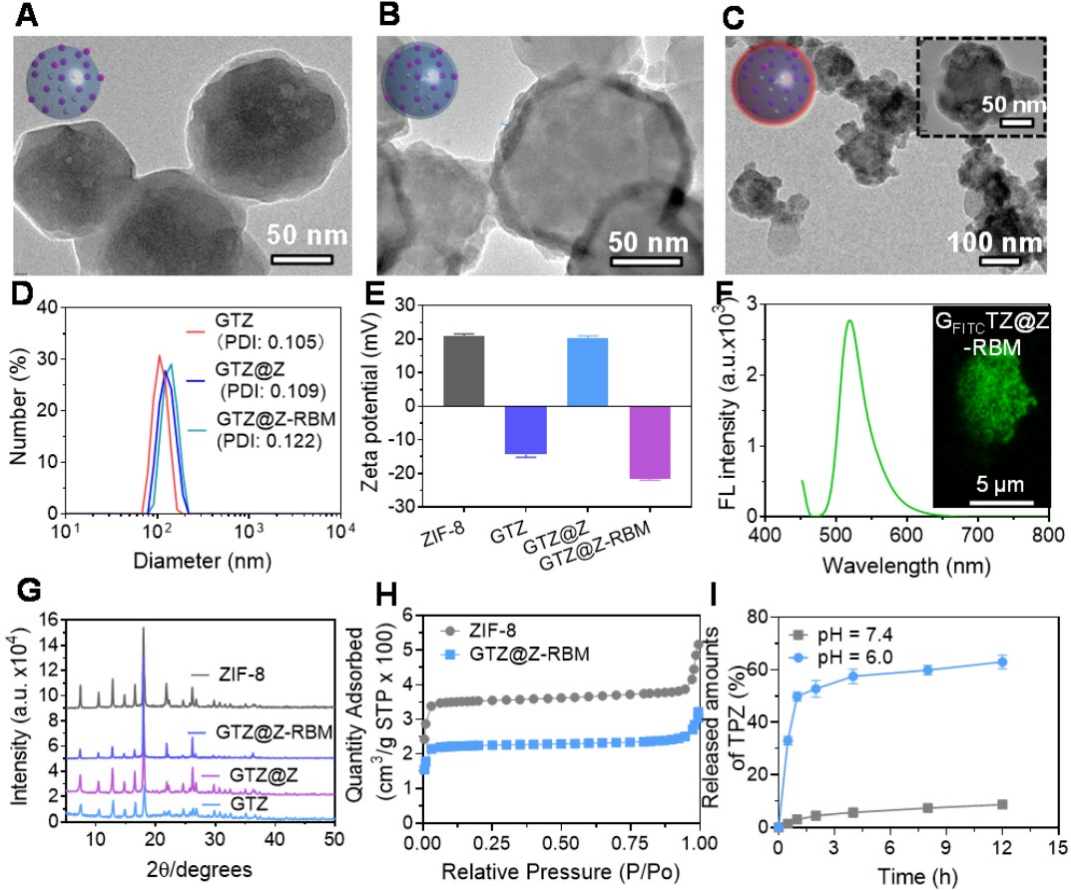
** Characterization of GTZ@Z-RBM NPs.** TEM images of (**A**) GTZ NPs, (**B**) GTZ@Z NPs, and (**C**) GTZ@Z-RBM NPs. (**D**) Recorded hydrodynamic sizes and (**E**) Zeta potential of GTZ, GTZ@Z, and GTZ@Z-RBM measured by dynamic light scattering (DLS). (**F**) Fluorescence spectrum of G_FITC_TZ@Z-RBM NPs, inset shows the confocal laser scanning microscopy (CLSM) image of G_FITC_TZ@Z-RBM NPs. (**G**) XRD patterns of ZIF-8, GTZ, GTZ@Z, and GTZ@Z-RBM. (**H**) N_2_ isotherms of ZIF-8 and GTZ@Z. (**I**) TPZ released from GTZ@Z NPs at pH = 7.4 and 6.0 in PBS.

The GTZ@Z-RBM nanoreactors were prepared by co-precipitation and epitaxial growth process under mild conditions (*i.e.*, in water, at room temperature) as described in previous reports 43, 44. GOx and TPZ were first embedded into ZIF-8 NPs (defined as GTZ) by the one-pot encapsulation approach. The typical transmission electron microscope (TEM) image of as-synthesized ZIF-8 NPs showed uniform morphology and narrow size distribution with a diameter of ~80 nm (**Figure S1 and S2**). After co-precipitation with GOx and TPZ, the size of NPs slightly increased from 80 to 100 nm with the incorporation of the enzyme and TPZ recorded by TEM and dynamic light scattering (DLS). The prepared GTZ NPs showed uniform distribution with a diameter of ~100.7 nm and particle dispersion index (PDI) of 0.105 (**Figure 2A**). Next, the loading efficiency of GOx was calculated to be about 95% (~376.9 µg mg^-1^, 27.3 wt%) using fluorescein isothiocyanate (FITC)-labeled enzyme according to the standard calibration curve based on the fluorescence spectra at 520 nm of the difference between the initial solution and supernatant collected after centrifugation (**Figure S3**). Compared to the previous studies (**Table S1**), high loading capacity of GOx was achieved in our system. The encapsulation rate of TPZ in the GTZ was about ~11.2%, as determined by UV-vis analysis using the standard calibration curve (**Figure S4**). Subsequently, another ZIF-8 shell was coated on the GTZ NPs to form the core-shell structure (GTZ@Z) by the epitaxial growth process. TEM images of NPs in** Figure 2B** verified the shell structure of ZIF-8 coats on the surface of GTZ NPs with ~8.7 nm thickness (109.4 nm with PDI of 0.109). The biocompatibility of GTZ@Z was further improved by sonication with RBM to fabricate a biomimetic nanoreactor defined as GTZ@Z-RBM. For this, the RBCs were collected and squeezed through porous membranes to obtain RBM vesicles (**Figure S5),** which were cloaked onto GTZ@Z NPs by sonication. As shown in **Figure 2C**, a faint dense corona surrounding the surface of NPs was observed, indicating that RBM was coated onto the GTZ@Z. DLS analysis showed a ~10.7 nm increase in the hydrodynamic diameter of GTZ@Z NPs upon RBM coating (~120.1 nm with PDI of 0.122) (**Figure 2D**), consistent with the reported RBM thickness 45. In addition, the GTZ-RBM NPs were synthesized by the same processes (**Figure S6**).

The stability of GTZ@Z-RBM nanoreactors was measured in fetal bovine serum (10%) for 24 h, and the size was found to be very stable compared to GTZ@Z NPs (**Figure S7**).** Figure 2E** shows ζ-potential measurements of ZIF-8, GTZ, GTZ@Z and GTZ@Z-RBM as +20.83 mV, -14.40 mV, +20.33 mV, and -21.63 mV, respectively, confirming the formation of GTZ@Z-RBM nanoreactors. The negative ζ-potential of GTZ indicated that the part of the loaded GOx was exposed on the surface of NPs. After coating with the ZIF-8 shell, the opposite ζ-potential of GTZ@Z revealed that the GOx was completely wrapped in the NPs, and the more negative ζ-potential following RBM modification indirectly demonstrated the successful synthesis of GTZ@Z-RBM.

FITC-labeled GOx (GOx_FITC_) was introduced into the nanoreactor forming G_FITC_TZ@Z-RBM to confirm that GOx was indeed encapsulated in ZIF-8. As is evident from the fluorescence spectrum (**Figure 2F**), G_FITC_TZ@Z-RBM suspension yielded green fluorescence at 519 nm with excitation at 494 nm. The CLSM image (inset of **Figure 2F**) showed uniform green fluorescence of NPs, further verifying that the GOx was indeed embedded in ZIF-8. Furthermore, TEM images of GTZ after calcination showed small cavities (**Figure S8**), demonstrating encapsulation of GOx and TPZ in ZIF-8 rather than adsorption on the surface of NPs.

The powder X-ray diffraction (PXRD) patterns verified the crystal structure of GTZ@Z-RBM NPs similar to the pure ZIF-8, indicating that embedded GOx and TPZ negligibly influenced the crystallinity of ZIF-8 (**Figure 2G**). Also, the results of nitrogen adsorption-desorption isotherm assay showed that the Brunner-Emmet-Teller (BET) surface area of GTZ@Z (1027.2 m^2^ g^-1^) was smaller than pure ZIF-8 (1710.4 m^2^ g^-1^), which demonstrated the presence of TPZ and GOx in the NPs (**Figure 2H**). These results demonstrated the successful synthesis of GTZ@Z-RBM using mild condition.

Furthermore, the drug release behavior of GTZ@Z-RBM was investigated in different pH solutions containing 5 mM glucose. Upon exposure to an acidic environment (pH=6.0), GTZ@Z-RBM NPs displayed a burst release of TPZ after 2 h, and more than 63% of the drug was released within 12 h (**Figure 2I**). Comparatively, less than 8% drug was released at pH=7.4. Therefore, GTZ@Z-RBM NPs can be used as an acid pH-responsive drug delivery system for on-demand drug release.

### *In Vitro* biological properties of GTZ@Z-RBM

**Figure 3:**
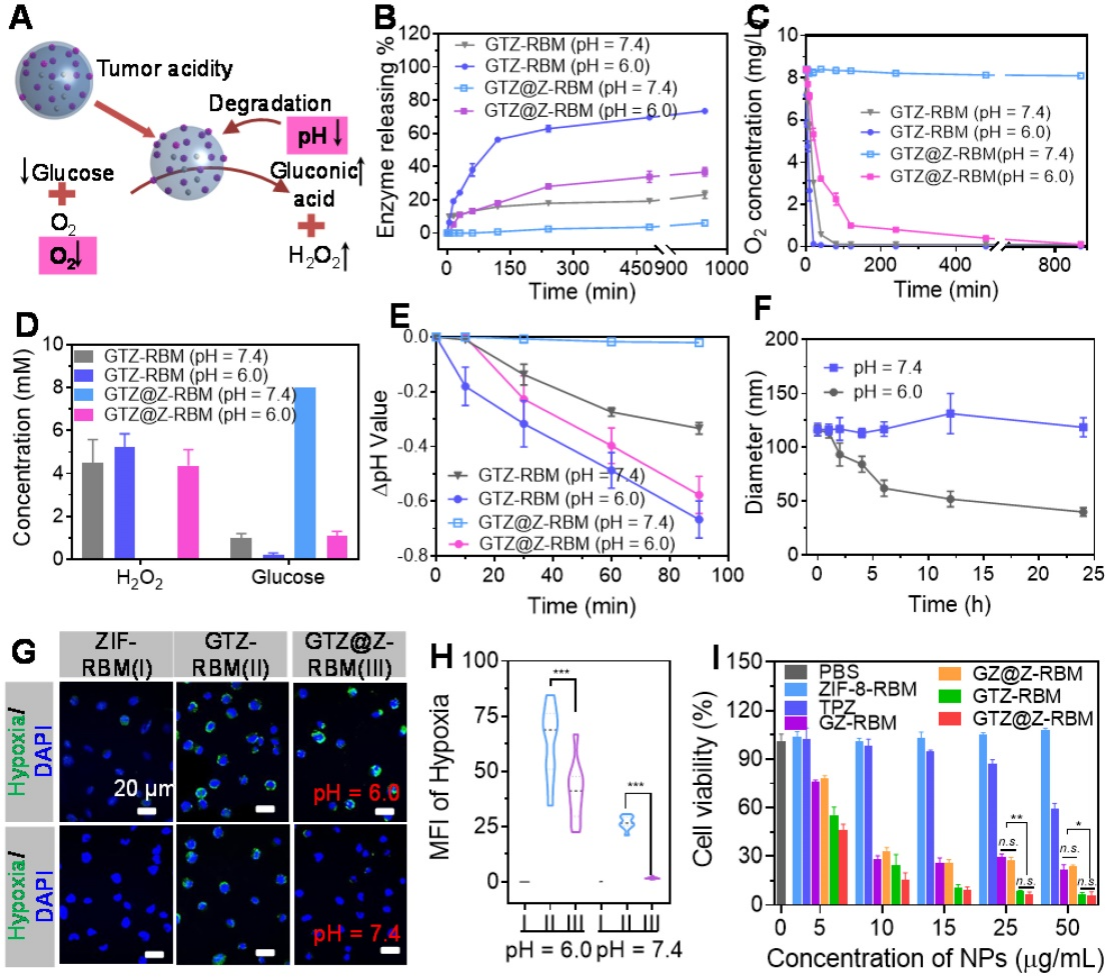
***In Vitro* biological properties of GTZ@Z-RBM. (A)** Schematic illustration of biological properties of nanoreactor under acidic condition.** (B)** FITC-labeled GOx releasing property of GTZ-RBM and GTZ@Z-RBM NPs in 5 mM glucose at pH = 7.4 and 6.0. **(C)** Oxygen consumption variations of GTZ-RBM and GTZ@Z-RBM NPs in 5 mM glucose at pH = 7.4 and 6.0. **(D)** Concentration of generated H_2_O_2_ and remnant glucose after treatment of GTZ and GTZ@Z NPs in 5 mM glucose at pH = 7.4 and 6.0. **(E)** Change in pH value GTZ-RBM and GTZ@Z-RBM NPs in 5 mM glucose at pH = 7.4 and 6.0. **(F)** Size changes of GTZ@Z-RBM NPs in 5 mM glucose at pH = 7.4 and 6.0. **(G)** Fluorescence confocal images of cellular hypoxia of 4T1 cells after treatment with PBS (I), GTZ-RBM (II), and GTZ@Z-RBM (III) in DMEM at pH = 7.4 and 6.0. **(H)** Quantification of hypoxia-positive mean fluorescence intensities (MFI) of 4T1 cells after treatment with PBS (I), GTZ-RBM (II), and GTZ@Z-RBM (III) in DMEM at pH = 7.4 and 6.0. **(I)** Viability of 4T1 cells after treatment with PBS, ZIF-8-RBM, TPZ, GZ-RBM, GZ@Z-RBM, GTZ-RBM, or GTZ@Z-RBM at pH = 6.0.

Subsequently, the biocatalytic properties of GTZ@Z-RBM nanoreactor were activated under acidic conditions (**Figure 3A**). First, to verify the “ON/OFF” enzymes releasing from GTZ@Z-RBM NPs, fluorescently labeled G_FITC_TZ@Z-RBM NPs were investigated in PBS at pH = 6.0 and 7.4. **Figure 3B** shows that GTZ@Z-RBM NPs released the enzyme with ~36.8% under acidic conditions, while enzyme release was only ~6.06% at physiological pH = 7.4 after 16 h, displaying acid-responsive enzymes release performance. On the contrary, the enzymes release was ~23.2% from GTZ-RBM NPs at neutral conditions. Moreover, the GOx release behavior of GTZ@Z-RBM nanoreactors with different thickness of ZIF-8 shells was studies in physiological pH, and the results determined that the ZIF-8 shell could well prevent enzyme release in physiological pH (**Figure S9**). Meanwhile, the O_2_ concentration rapidly decreased from 8.4 to 0.593 mg L^-1^ after treatment of GTZ-RBM NPs at pH 7.4 for 40 mins, while it negligibly decreased after treatment of GTZ@Z-RBM NPs. (**Figure 3C**). As shown in **Figure 3D**, no toxic H_2_O_2_ was produced by GTZ@Z-RBM NPs at pH = 7.4, and their catalytic activity was shielded at neutral condition, suggesting that the toxicity of GTZ@Z-RBM NPs was lower than GTZ-RBM NPs. On the contrary, glucose could be consumed by the GTZ@Z-RBM NPs at pH = 6.0, indicating that this nanoreactor could be used to catalyze glucose for cancer starvation therapy. Meanwhile, pH value of glucose solution was almost stable after treatment with GTZ@Z-RBM NPs at pH = 7.4 (**Figure 3E**), but decreased at pH = 6.0, indicating that glucose oxidation was accelerated following the decomposition of nanoreactor and the release of GOx and TPZ. Due to the pH-responsive behavior of the nanoreactor, the decomposed structure was observed upon exposure to an acidic environment for 24 h (**Figure S10**), and the size of NPs decreased from ~120 nm to ~40 nm under acidic conditions (**Figure 3F**).

A series of cell experiments were carried out to investigate the* in vitro* biological behavior of GTZ@Z-RBM NPs. FITC-labeled GTZ@Z-RBM NPs were incubated with 4T1 cells followed by quantitative analysis with flow cytometry to verify NPs uptake by endocytosis after 4 h (**Figure S11**). The CLSM images of 4T1 cells incubated with G_FITC_TZ@Z-RBM NPs at different time points showed increased fluorescence intensity (**Figure S12**), consistent with flow cytometry results. Subsequently, RAW264.7 murine macrophages were incubated with G_FITC_TZ@Z-RBM NPs to evaluate the immune-evading ability of NPs. A bright green fluorescence was observed in RAW264.7 cells treated with G_FITC_TZ@Z NPs, while there was only a dim green fluorescence in RAW264.7 cells treated with G_FITC_TZ@Z-RBM NPs (**Figure S13**), indicating the RBM could improve the biocompatibility and immune-escaping ability of the nanoreactor.

Next, PBS, GTZ-RBM, and GTZ@Z-RBM NPs were incubated with 4T1 cells at pH = 7.4 and 6.0. Cell hypoxia was characterized by CLSM using a hypoxia probe, pimonidazole hydrochloride. A strong green fluorescence (hypoxia-positive signal) was observed in 4T1 cells after treating with GTZ-RBM and GTZ@Z-RBM NPs in DMEM at pH = 6.0 (**Figure 3G**). However, a negligible green fluorescence signal was visible after treatment with GTZ@Z-RBM NPs at pH = 7.4, demonstrating that our designed nanoreactor had pH-responsive behavior at the cellular level. The corresponding hypoxia MFI was further quantified by the Image J software (**Figure 3H**). The generated concentration at H_2_O_2_ at the cellular level was consistent with the result in **Figure 3C** (**Figure S14**). The 2',7'-Dichlorodihydrofluorescein diacetate (DCFH-DA) could be oxidized to (2',7'-dichlorofluorescein) DCF by H_2_O_2_ accompanied with green fluorescence observed after treating the cells with GTZ@Z-RBM NPs at pH = 6.0 (**Figure S15**). To evaluate the cytotoxicity of H_2_O_2_ in 4T1 cells, cellular viability was decreased rapidly with an increased concentration of H_2_O_2_ (**Figure S16**) due to its genotoxic and cytotoxic effects inducing DNA damage and apoptosis. Immunofluorescence results from γ-H2AX expression showed that GTZ@Z-RBM induced DNA damage (**Figure S17**). These results indicated that the GTZ@Z-RBM NPs could be used to consume glucose, aggravate hypoxia, and produce toxic H_2_O_2_.

Furthermore, the cytotoxicity of ZIF-8-RBM, TPZ, GZ-RBM, GZ@Z-RBM, GTZ-RBM, and GTZ@Z-RBM was evaluated by the standard MTT cellular viability assay at different pH values (7.4 and 6.0). At neutral pH, increasing concentrations of NPs (ZIF-8-RBM, GZ@Z-RBM, and GTZ@Z-RBM) showed no apparent toxicity to 4T1 cells after 48 h (**Figure S18**); however, cellular viability decreased rapidly in the presence of GZ-RBM and GTZ-RBM, indicating that toxic H_2_O_2_ was generated at neutral pH without the ZIF-8 shell. Compared to free TPZ, an enhanced anticancer effect was observed after treatment with GTZ@Z-RBM upon exposure to an acidic environment (**Figure 3I**). In addition, calcein-AM and propidium iodide (PI) were stained to the living and dead cells, respectively. Flow cytometry results and CLSM images were consistent with the MTT assay (**Figure S19 and S20**), suggesting that the pH-responsive GTZ@Z-RBM NPs can be used for starvation therapy and activating the hypoxia drug through depleting glucose and consuming O_2_. The combination index (CI) was calculated according to the formula presented in Supplementary Material, and the CI was 0.56, indicating a synergetic effect of GOx and TPZ.

### *In vivo* biological properties of GTZ@Z-RBM

**Figure 4:**
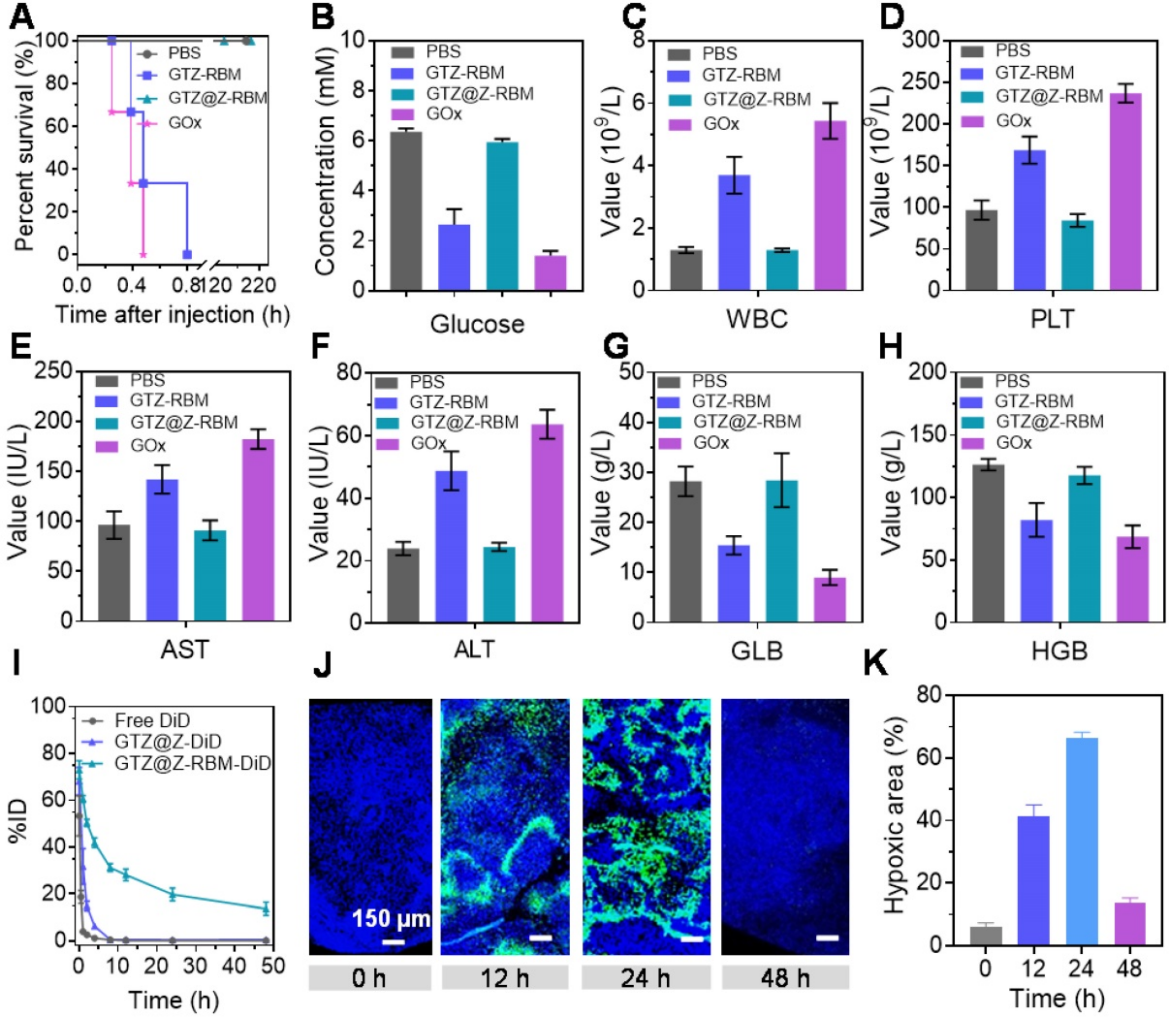
***In vivo* biological properties of GTZ@Z-RBM:** (**A**) Cumulative survival rate of mice (n = 3 per group) after *i.v.* injection with PBS, GTZ-RBM, GTZ@Z-RBM, or GOx. Hematology evaluations including (**B**) blood glucose concentration, (**C**) WBC, (**D**) PLT, (**E**) AST, (**F**) ALT, (**G**) globulin, and (**H**) hemoglobin. (**I**) Blood circulation lifetime of DiD, GTZ@Z-DiD, and GTZ@Z-RBM-DiD after *i.v*. injection into mice (n = 3 per group). (**J**) Representative immunofluorescence images of tumor slices stained with the hypoxia probe kit at post-injection of GTZ@Z-RBM NPs (0, 12, 24, and 48 h). (**K**) Quantification of tumor hypoxia positive areas for different treatment groups, Data are shown as mean ± SEM (n = 3).

We further investigated the *in vivo* biological behavior of GTZ@Z-RBM. First, the biological safety of nanoreactors was studied through survival experiments and hematological parameters of mice following different treatments. The healthy mice were *i.v*. injected with PBS, GTZ-RBM, GTZ@Z-RBM, or GOx to investigate the* in vivo* biocompatibility. The mice treated with free GOx and GTZ-RBM died within 0.4 and 0.8 h (**Figure 4A**), respectively, because of toxic H_2_O_2_ generated by exposure to GOx in the blood. On the contrary, the mice *i.v.* injected with GTZ@Z-RBM survived for more than one week, indicating good biosecurity of GTZ@Z-RBM. As shown in **Figure 4B**, the blood glucose concentration in the dead mice was sharply decreased to 2.6 and 1.4 mM.

We further elucidated the mechanisms by which GTZ@Z-RBM could avoid systemic toxicity. We studied the hematological parameters of the mice after treatment with PBS, GTZ-RBM, GTZ@Z-RBM, or GOx. The mice treated with GOx and GTZ-RBM showed significantly higher numbers of platelets (PLT) and white blood cells (WBC) than the mice treated with other groups (**Figure 4C and 4D**), indicating hematotoxicity and inflammation in mice due to excess poisonous H_2_O_2_. Also, GOx and GTZ-RBM groups suffered acute inflammation and damage to the liver as evidenced by high levels of aspartate aminotransferase (AST) and alanine aminotransferase (ALT) (**Figure 4E and 4F**). Besides, values of globulin (GLB) and hemoglobin (HGB) in GOx and GTZ-RBM groups were lower than others, revealing severe side effects from GOx and GTZ-RBM (**Figure 4G and 4H**). These results demonstrated that GTZ@Z-RBM, because of its ZIF-8 shell, could significantly reduce systemic toxicity from H_2_O_2_, generated by GOx.

Next, we used DiD as a common near-infrared probe to monitor the blood circulation lifetime of GTZ@Z-RBM. After the DiD was loaded into GTZ@Z and GTZ@Z-RBM (defined as GTZ@Z-DiD and GTZ@Z-RBM-DiD), the fluorescence signals of free DiD, GTZ@Z-DiD, and GTZ@Z-RBM-DiD in the blood were detected at different time points. Compared with free DiD and GTZ@Z-DiD, GTZ@Z-RBM-DiD showed a prolonged half-life in blood circulation. Meanwhile, the area under the curve (AUC) of GTZ@Z-RBM-DiD was significantly increased in blood compared to the free DiD (**Figure 4I**). And then, the fluorescence signals in tumor xenografts were also detected to evaluate *in vivo* biodistribution profiles of GTZ@Z-RBM-DiD at different time points. As shown in **Figure S21**, the DiD fluorescence in tumor increased with time post *i.v.* injection, indicating enhanced accumulation and retention of NPs in the tumor. The fluorescence intensity of DiD continually increased at the tumor site over the first 12 h, and then slowly got weaker (**Figure S22**). The major organs and the tumor were collected from sacrificed mice at 48 h post-injection. The GTZ@Z-RBM-DiD fluorescence signal was detected in the tumor, further confirming its strong ability of tumoral accumulation (**Figure S23 and S24**).

Next, the deoxygenation ability of GTZ@Z-RBM *in vivo* was determined by immunofluorescence imaging at different time points post-injection of NPs. The tumors were surgically excised after pimonidazole administration, immune-stained with FITC-Mab1. The series of tumor slices were recorded by Tissue FAXSi imaging. Significant hypoxic fluorescence was observed 24 h post-injection of NPs (**Figure 4J**). The hypoxic fluorescence gradually increased at 24 h after injection with GTZ@Z-RBM, suggesting that the O_2_ in the tumors was consumed by GOx rapidly (**Figure 4K**). These results indicated that GTZ@Z-RBM efficiently exhausted the O_2_ in the tumor after *i.v.* injection, resulting in enhanced tumor deoxygenation.

### *In vivo* cancer therapy and suppression of tumor metastasis

**Figure 5:**
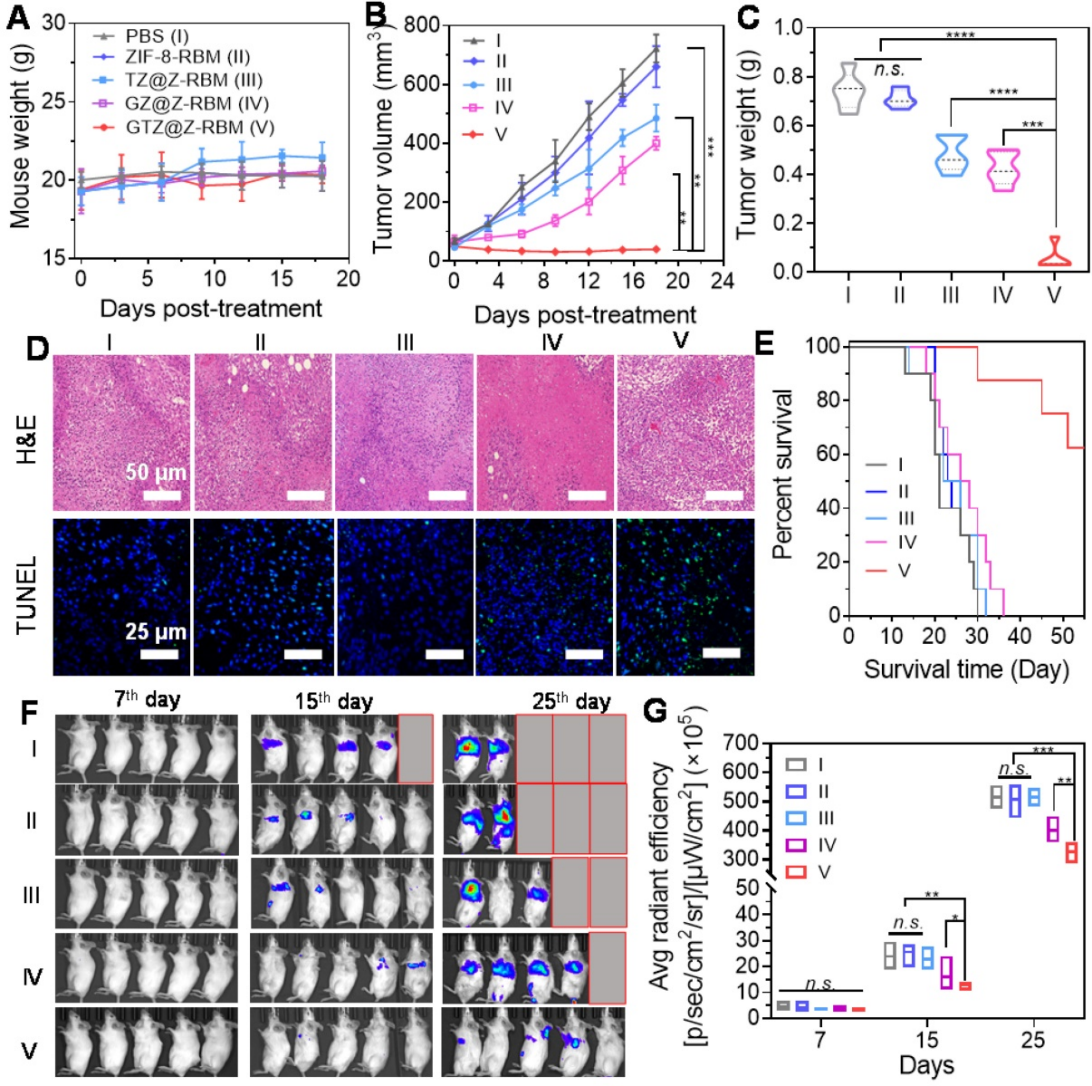
***In vivo* cancer therapy and suppression of tumor metastasis.** (**A**) Weight of mice (n = 5) treated with different formations. (**B**) Tumor volume changes of mice (n = 5) injected with different formations. (**C**) Tumor weight of mice on day 18 post treatment. (**D**) Representative H&E and TUNEL staining images of tumor tissues after different treatments. (**E**) Morbidity-free survival of mice with metastatic 4T1 tumors after various treatments to eliminate primary tumors. (**F**) *In vivo* bioluminescence images and (**G**) Quantitative analysis to track the spreading and growth of* i.v.* injected 4T1-luc cancer cells in different groups of mice after various treatments. Various groups are indicated: PBS (I), ZIF-8-RBM (II), TZ@Z-RBM (III), GZ@Z-RBM (IV) and GTZ@Z-RBM (V).

*In vivo* multimodal synergistic therapy effect of GTZ@Z-RBM was measured in a breast tumor model. The 4T1 tumor-bearing mice, randomly divided into five groups (n = 5 mice per group with the initial tumor size of ~100 mm^3^), were received *i.v.* injections of PBS (I), ZIF-8-RBM (II), TZ@Z-RBM (III), GZ@Z-RBM (IV), or GTZ@Z-RBM (V). The body weight of treated mice (**Figure 5A**) showed no significant changes during different administrations, suggesting no apparent toxicity. In the tumor growth profiles (**Figure 5B**), the tumor size of PBS and ZIF-8-RBM control groups increased significantly. By contrast, the tumor size of the GTZ@Z-RBM group shrank gradually to ~39.6 mm^3^ within 18 days after *i.v.* injection. The tumor growth inhibition of TZ@Z-RBM group was achieved by chemotherapy alone (~32.8% of growth inhibition) compared with the PBS group because of the internal tumor hypoxia-activating toxicity of TPZ. The tumor growth inhibition by starvation alone was ~44.6% due to the intra-tumoral glucose depletion by GZ@Z-RBM. The strongest anti-tumor effect was observed in GTZ@Z-RBM group, showing almost complete elimination of the tumors (~94.6% of growth inhibition). Meanwhile, the weight and images of tumors from sacrificed mice on day 18 further verified that GTZ@Z-RBM treatment was the most effective in suppressing tumor growth (**Figure 5C and S25**). The tumors were further used for H&E and TUNEL staining (**Figure 5D**), confirming that GTZ@Z-RBM generated the most damage to tumors among all groups. Finally, the heart, lung, liver, kidney, and spleen were collected for histological examination (**Figure S26**). The H&E staining confirmed *in vivo* biosafety and biocompatibility of various treatments.

Next, 4T1 cells with stable luciferase expression (4T1-luc) were used to establish the metastatic breast cancer model by* i.v.* injection. The mice were closely monitored after various treatments to evaluate the therapeutic outcomes of GTZ@Z-RBM based on tumor starvation, high concentration of toxic H_2_O_2_, and deoxygenation-activated chemotherapy. Six of ten mice receiving *i.v.* injection of 4T1-luc cells survived for 55 days after treatment with GTZ@Z-RBM in marked contrast to mice in the other four groups that all died within 30-40 days (**Figure 5E**). The proliferation and growth of 4T1-luc cancer cells in various groups could be monitored by bioluminescence imaging (**Figure 5F**). Quantitative analysis of bioluminescence images showed insignificant cancer metastasis 7 days after *i.v.* injection with 4T1-luc cells (**Figure 5G**). Mice were *i.v.* injected on the following day with PBS, ZIF-8-RBM, TZ@Z-RBM, GZ@Z-RBM, or GTZ@Z-RBM. Subsequently, the images captured on day 15 and 25 exhibited strong bioluminescence signals in the PBS, ZIF-8-RBM, and TZ@Z-RBM groups, verifying metastatic spread. The bioluminescence signals were also detected in the GZ@Z-RBM group but the GTZ@Z-RBM group had almost no bioluminescence signals, conforming its superior therapeutic efficacy in inhibiting metastasis. These results provided evidence that multimodal synergistic therapy could effectively inhibit breast metastasis based on GOx-mediated tumor starvation, high concentration of toxic H_2_O_2_, and the subsequent deoxygenation-activated chemotherapy.

## Conclusion

In summary, we have successfully synthesized the core-shell structure GTZ@Z-RBM by co-precipitation and epitaxial growth process under mild conditions. We demonstrated that the enzyme and drug moleculars could be effectively encapsulated in the nanocarriers preventing enzymatic reaction under normal physiological conditions while promoting it in an acid environment. The GTZ@Z-RBM showed superior therapeutic efficacy based on GOx-mediated tumor starvation, toxic H_2_O_2_ generation, and the subsequent deoxygenation-activated chemotherapy. Most significantly, the breast cancer metastasis could be effectively inhibited by GTZ@Z-RBM.

## Supplementary Material

Supplementary methods and figures.
