# Supplementary material for: Rational design of non-toxic GOx-based biocatalytic nanoreactor for multimodal synergistic therapy and tumor metastasis suppression
Source: Theranostics. 2021 Oct 25;11(20):10001–11. doi: 10.7150/thno.65399 (PMC8581412; doi:10.7150/thno.65399)
Supplement: Supplementary file 1 — Supplementary methods and figures. [file thnov11p10001s1.pdf]

Rational design of non-toxic GOx-based biocatalytic nanoreactor for multimodal synergistic therapy and tumor metastasis suppression

Lifeng Hang[1*#], *Tao Zhang*[2#], *Hua Wen*[1#], Meng Li[1], *Lianbao Liang*[1], *Xinfeng Tang*[3], *Chunze Zhou*[4], *JunZhang Tian*[1], *Xiaofen Ma*[1], *Guihua Jiang*[1*]

[1] The Department of Medical Imaging, Guangdong Second Provincial General Hospital, Guangzhou, 518037, P. R. China
[2] Key Laboratory of Materials Physics, Institute of Solid State Physics, HFIPS, Chinese Academy of Sciences, Hefei, 230031, P. R. China
[3] Division of Molecular Medicine, Hefei National Laboratory for Physical Sciences at Microscale, School of Life Sciences, University of Science and Technology of China, Hefei, 230001, P. R. China
[4] Interventional Radiology Department, The First Affiliated Hospital of USTC, Division of Life Sciences and Medicine, University of Science and Technology of China, Hefei, 230001, P. R. China

[#] These authors contributed equally to this work.

Experiment section

Synthesis of GOx_{FITC}

GOx (10 mg) was treated with fluorescein isothiocyanate (FITC, 0.6 mg, 20 eq) in 50 mM borate buffer (pH 8.5, 2 mL) for 1 h at 25 ℃. Excessive FITC was removed *via* dialysis against in PBS at pH 7.4. The conjugation efficiency of GOx_{FITC} was quantified by the FITC/GOx molar ratio of the GOx_{FITC} conjugate. The concentration of GOx and FITC was quantified by UV absorbance (280 nm) and fluorescence intensity (excitation at 488 nm, emission at 514 nm), respectively.

Preparation of RBM

RBC membranes (RBM) were extracted following previously published protocols with modifications. RBCs were washed with 1 × PBS in ice and then treated with a hypotonic medium. The RBCs were suspended in 0.25× PBS for 40 min at 4 ℃, and then were centrifuged (9000 rpm, 5 min, 4 ℃). The precipitate of pink i-RBCs was collected for further use. The RBC suspension was sonicated with a microtip using VibraCell VCX 130 (Sonic and Material, Inc., Newtown, CT, USA) (20 kHz, 130 W, 5 min). The obtained RBM suspension was further extruded through 1 μm, 400 nm, and then 200 nm polycarbonate membranes (Whatmann, Maidstone, UK) using an Avanti mini extruder device (Avanti Polar Lipids, Inc., Alabaster, AL, USA) for 11 times, respectively.

Loading Efficiency of TPZ and GOx

The payload of GOx was determined *via* FITC labeled by using the standard calibration curve based on the fluorescence spectra at 520 nm of the difference

between the supernatant collected after centrifugation and initial solution. Meanwhile, loading amount of TPZ was obtained by using the standard calibration curve based on the UV−vis absorption intensity at 470 nm of the difference between the supernatant collected after centrifugation and initial solution.

In addition, the GTZ NPs were calcinated at 250 ^{o}C for 2 h under nitrogen. The calcinated GTZ NPs were characterized by TEM.

Enzyme releasing and catalytic ability measurement

1 mg FITC labeled GTZ-RBM and GTZ@Z-RBM were dispersed into 1 mL of glucose solution (5 mM) at 7.4 and 6.0. The enzyme labeled with fluorescent molecules was released from NPs and recorded by fluorophotometer. The real time pH values and dissolved O_2 of the solutions were respectively measured with a pH meter and oxygen detector.

Detection of H_2O_2

1 mg of GTZ-RBM and GTZ@Z-RBM were respectively added into 1 mL of glucose solution (5 mM) at 7.4 and 6.0 for 30 min, then 200 μL of ammonium hydroxide and 100 μL of $TiOSO_4 \cdot xH_2SO_4 \cdot xH_2O$ solution (5% wt) was added into the above solution to from the precipitate for 10 min. After that, the precipitate was collected and dissolved with 200 μL of H_2SO_4 solution (2 M). Finally, homogeneous solution was recorded by the UV-Vis spectrometer.

TPZ releasing measurement

The drug release experiments were divided into two groups: (1) pH 7.4; (2) pH 6.0 phosphate buffered saline (PBS 10 mM). GTZ@Z-RBM (1 mg mL^{-1} 5 mL) was

dispersed into 5 mL PBS at different pH under stirring at 37 ℃ in the capped vials. At predetermined time intervals, 200 μL of the buffer solution was removed for UV-vis spectrum measurement of TPZ. The cumulative amounts of TPZ released were determined according to the UV-vis absorption intensity at 470 nm, using the standard calibration curve.

Cell Cultures

According to the recommended procedure, 4T1 cells were cultured in DMEM medium supplemented with 10% FBS and 1% penicillin/streptomycin under humidified normoxic (95% air, 5% CO_2) conditions at 37 ^{o}C.

Cellular uptake of GTZ@Z-RBM

Cell phagocytosis was assessed *via* flow cytometer and CLSM. To intuitively display the cellular uptake of GTZ@Z-RBM, FITC labelled GOx was introduced into ZIF-8. 4T1 cells with 50000 cells/well in 0.5 mL of DMEM were seeded in 24-well plates and incubated for 24 h at 37 ℃ in 5% CO_2 atmosphere. Then the cells were further incubated with G_{FITC}TZ@Z-RBM at 37 ℃ for 2, 4, and 8 h to evaluate the uptake. At last, flow cytometer and LCMS were utilized to detect the phagocytosis of NPs.

Hypoxia Measurements

4T1 cells were seeded on coverslips in a 24-well plate at a density of 5×10^4 cells per well and incubated overnight. Then the cells were incubated with fresh medium containing PBS, GTZ-RBM, and GTZ@Z-RBM at pH 7.4 or 6.5 for 4 h. The cells were incubated with fresh medium containing pimonidazole hydrochloride (60 μg/mL)

for another 1 h then fixed with paraformaldehyde, followed washed with PBS and stained with anti-pimonidazole mouse monoclonal antibody (FITC-Mab1, Hypoxyprobe-1^{TM} plus kit, Hypoxyprobe Inc.; Burlington, MA) and DAPI. The slides were observed by Zeiss LSM 880 confocal laser scanning microscope (Carl Zeiss, Oberkochen, Germany).

ROS Measurements

For ROS observations on fluorescence microscope, 4T1 cells were digested and resuspended into 24-well plates with about 50000 cells/well in 0.5 mL of DMEM, and incubated for 24 h at 37 ℃ in 5% CO_2 atmosphere. Subsequently, 100 mM non-fluorescent 2',7'-Dichlorofluorescin diacetate (DCFH-DA) containing GTZ-RBM and GTZ@Z-RBM NPs was added into DMEM with different pH, which could form the fluorescent matter DCF (green fluorescence)

DNA Damage

4T1 cells were seeded on coverslips in a 24-well plate at a density of 5×10^4 per well at 37 ℃ overnight. Cells were further divided into the following groups: PBS, H_2O_2 (1 mM), GTZ@Z-RBM (50 μg/mL), and GTZ@Z-RBM+CAT. All groups were then exposed to fresh medium with pH of 6.0. After incubation for 24 h, the cells were washed and fixed with 4% paraformaldehyde (PFA). Cells were incubated with anti-γ-H2AX mouse monoclonal antibody (Abcam Inc., Cambridge, MA) at a dilution of 1:1000 overnight at 4 ℃. Cells were then washed before staining with a Cy3-labeled secondary antibody for 60 min. Then, 2-(4-Amidinophenyl)-6-indolecarbamidine dihydrochloride (DAPI, Sigma Aldrich, St.

Louis, MO) was used to stain the cell nuclei. The γ-H2AX level was examined on a confocal laser scanning microscope (LSM 880 with Airyscan, Carl Zeiss, Jena, Germany).

Toxicity of H_2O_2

4T1 cells were seeded on coverslips in a 96-well plate at a density of 3000 per well at 37 ℃ overnight. Cells were incubated with different concentration of H_2O_2 (100, 50, 25, 12.5, 6.25, 3.125, 1.5625 μM) for 24 h. MTT solution (10 μL) was added to each well of the microtiter plate and the plate was incubated for additional 4 h. Subsequently, the MTT contained culture medium was replaced with 100 μL of DMSO. Absorbance values of formazan were determined with Bio-Rad model-680 microplate reader at 490 nm.

Live/Dead staining

To evaluate cell viability of cells after various treatments, 4T1 cells were stained using live/dead kit (Weikai, Tianjin, China) by CLSM (Zeiss LSM 880+Airyscan) and flow cytometer. 4T1 cells were seeded into a 24-well plate with a density of 2×10^4 cells/well. Six groups including: ZIF-8-RBM, TPZ, GZ-RBM, GZ@Z-RBM, GTZ-RBM, and GTZ@Z-RBM with pH of 7.4 and 6.0 were applied in live/dead staining. After 4 h of incubation, the cells were washed with the PBS, and 500 μL of fresh medium was added. After 6 h post-treatment with different group, the cells in four groups were incubated with calcein-AM and propidium iodide (PI) at 37 ℃ for 5 min.

Combination index (CI)

The combination index (CI) was calculated according to following formula:

$$CI=C(GOx1)/C(GOx2)+C(TPZ1)/C(TPZ2)$$

The C(GOx1: 0.38 μg/mL) and C(TPZ1:0.03 μg/mL) are half maximal inhibitory concentration (IC50) in GTZ@Z-RBM NPs. In addition, C(GOx2: 0.8 μg/mL) and C(TPZ2: 0.35 μg/mL) are IC50 of single treatment. The CI was 0.56 which indicated synergetic effect of GOx and TPZ.

Animals and Tumor Model

Healthy female Balb/c mice (20-25 g) were purchased from the Vital River Laboratories (Beijing, China), and all animals received were cared in compliance with the guidelines outlined in the Guide for the Care and Use of Laboratory Animals, and all procedures were approved by the Guangdong Second Provincial General Hospital Animal Care and Use Committee. To obtain a xenograft model, the mice were injected subcutaneously with 1×10^5 4T1 cells (100 μL, 10% Matrigel) (BD Bioscience, Franklin Lakes, NJ).

Fluorescence Imaging of Biodistributin and Tumor Accumulation of GTZ@Z-RBM

Female Balb/c mice bearing 4T1 xenografts were intravenously injected with DiDlabeled GTZ@Z-RBM. The dose of DiD was 0.5 mg per kg mouse body weight. At the preset times, in vivo fluorescence images were acquired on the Xenogen IVIS Lumina system (Caliper Life Sciences, Alameda, CA). Moreover, at 48 h post-injection, the organs of mice including heart, lung, liver, spleen, kidney, and tumor were collected. Then fluorescence images were also acquired on the Xenogen

IVIS Lumina system as well. The parameters of mice fluorescence images via Xenogen IVIS Spectrum was listed below, including exposure time 0.5 s, emission filter 680 nm,excitation filter: 640 nm,filter position: 13, binning factor: 8, binning factor: 8,field of view: 22.5, and measured temperature -90 ℃.

Hematological Evaluations

The Balb/c mice (n = 5 per group) were *i.v.* injected with PBS, GOx, GTZ-RBM, and GTZ@Z-RBM (dose of 200 U/kg GOx, and 2 mg/kg TPZ) for 2 h. Blood from the angular vein of the eye was collected for hematology analysis, blood routine examination and blood biochemical analysis. The death of the mouse was also recorded.

Tumoral hypoxia

Mice bearing 4T1 tumors were i.v. injected with GTZ@Z-RBM (.dose of 200 U/kg GOx, and 2 mg/kg TPZ), and tumoral hypoxia immunofluorescence staining by pimonidazole hydrochloride (60 mg kg^{-1}) at different time (0, 12, 24, 48 h). Frozen tissue sections were stained with the mouse monoclonal antibody FITC-Mab1.

Immunohistochemical and Immunofluorescence Staining Analysis

The tumor tissues were fixed in 4% formaldehyde and embedded in paraffin. Subsequently, the tumor tissues were observed *via* H&E staining. Cell proliferation and apoptosis in tumor tissues were also analyzed by immunofluorescence staining of a terminal transferase dUTP nick-end labeling (TUNEL, Roche Diagnostics, Indianapolis, IN), respectively.

Statistical Analysis.

The statistical significance was assessed using Student's t-test (two-tailed); Significant differences between groups were indiCATed by *P < 0.05, **P < 0.01, and ***P < 0.001.

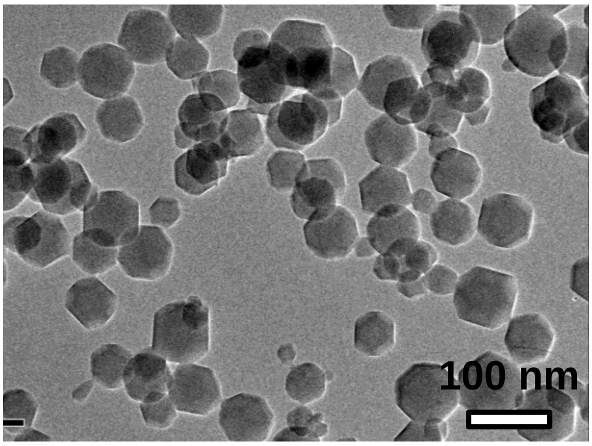

Fig. S1. TEM images of ZIF-8 NPs.

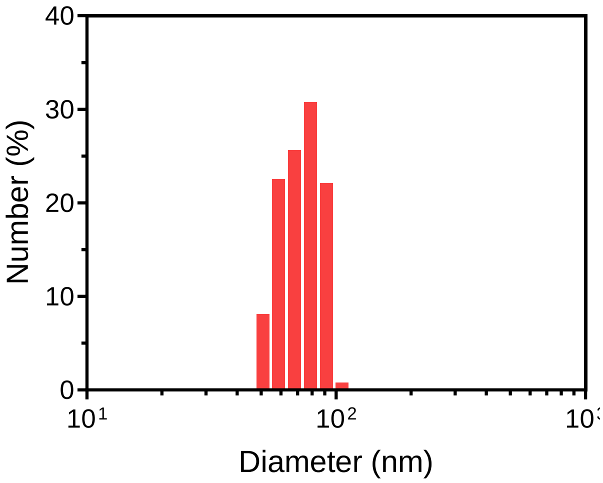

Fig. S2. Size distribution of ZIF-8 NPs.

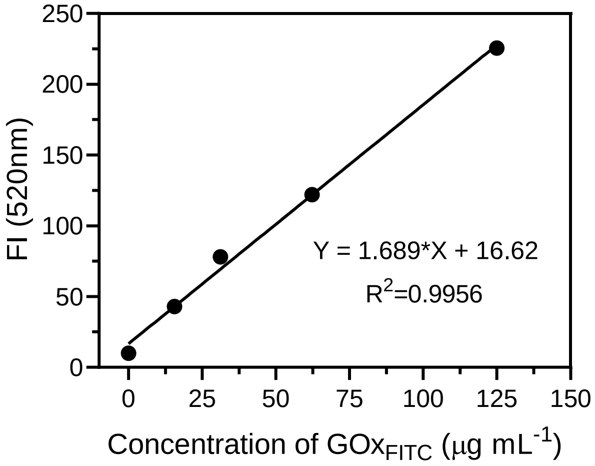

Fig. S3. Linear relationships between the fluorescence intensity at 520 nm and the concentration of GOx_{FITC}.

Loading efficiency of GOx	GOx loading capacity	GOx Wt%	References
90%	111 μg mg^{-1}	10	ACS Nano. 2018; 12: 10201-11
13.5%	/	/	ACS Nano. 2017; 11: 7006-18
/	65.8 μg mg^{-1}	6.2	Nat Catal. 2018; 1: 689-95
/	67.9 μg mg^{-1}	6.5	ACS Nano. 2018; 12: 7538-45.
**95%**	**376.9 μg mg^{-1}**	**27.3**	**In our work**

Table S1. The loading capacity of GOx in previous studies and our work.

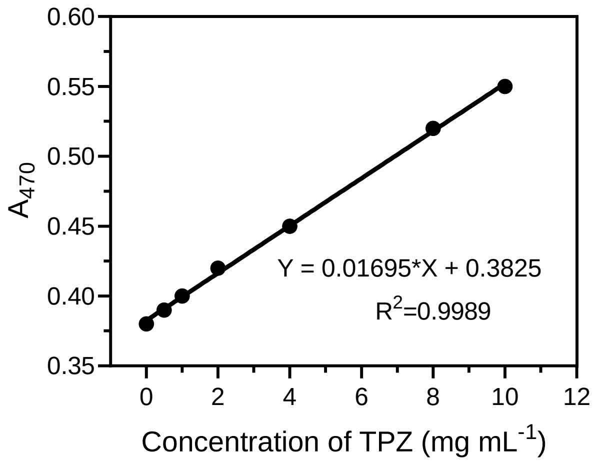

Fig. S4. Linear relationships between the UV-vis absorbance intensity at 470 nm and the concentration of TPZ.

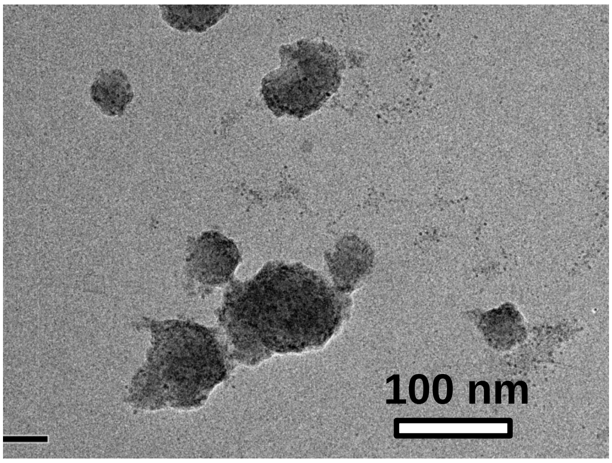

Fig. S5. TEM micrograph of erythrocyte membrane vesicles.

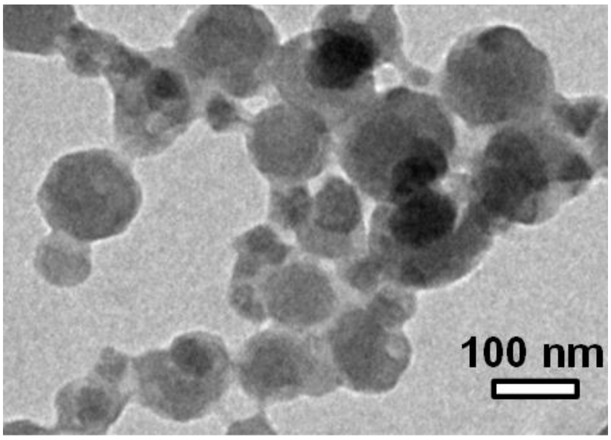

Fig. S6. TEM image of GTZ-RBM NPs.

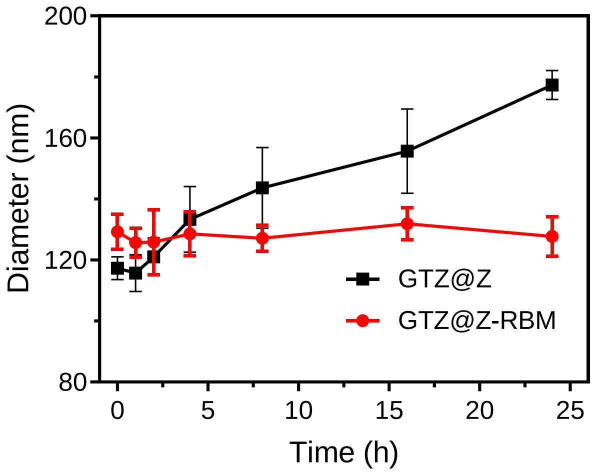

Fig. S7. Stability in 10% FBS solutions of GTZ@Z and GTZ@Z-RBM.

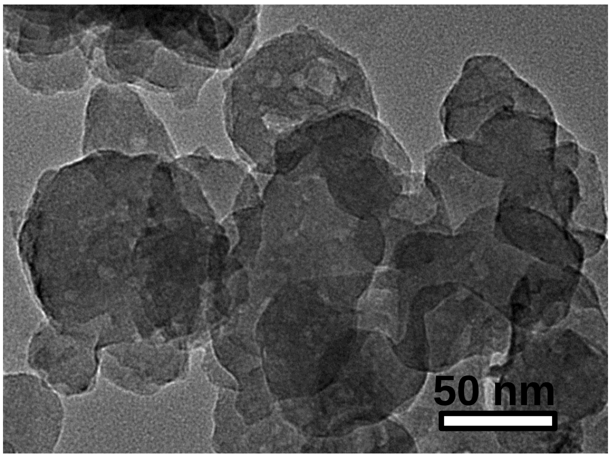

Fig.S8. TEM image of calcinated GTZ NPs.

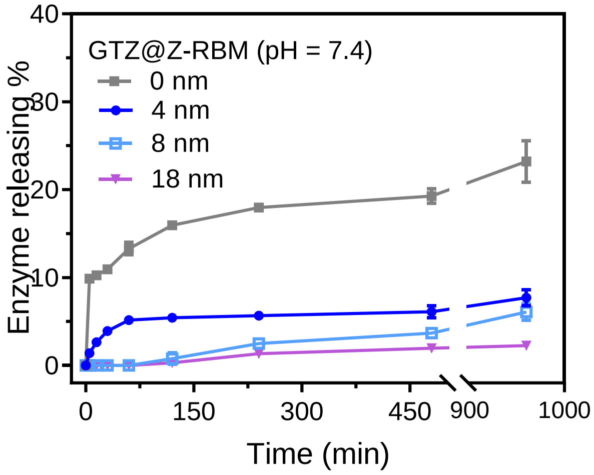

Fig. S9. The release behavior of GTZ@Z-RBM nanoreactors with different thickness of ZIF-8 shells was studies in physiological pH.

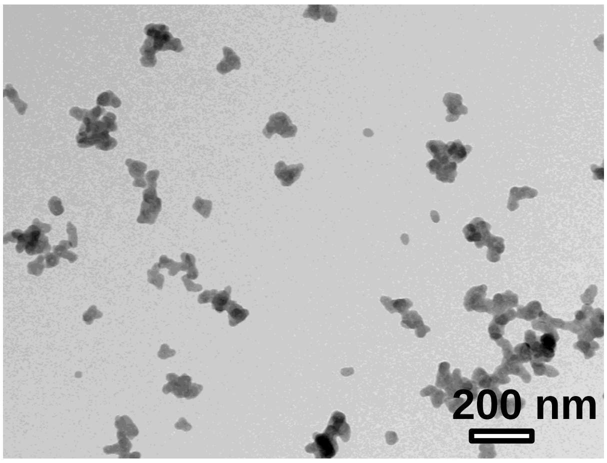

Fig. S10. TEM image of GTZ@Z-RBM NPs after treated with acidic condition for 24 h.

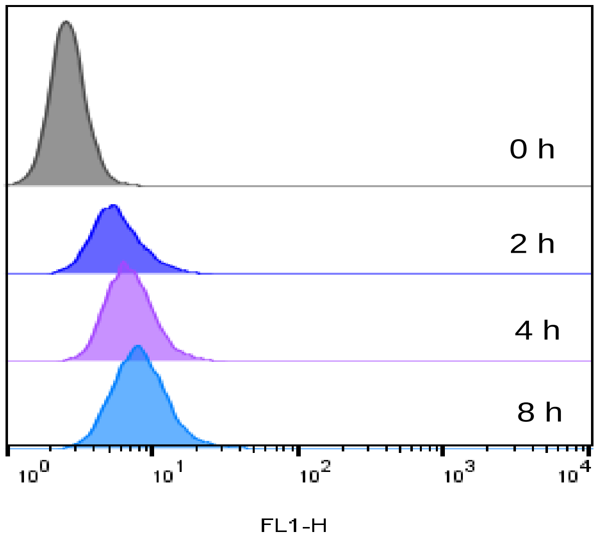

Fig. S11. Endocytosis of GTZ@Z-RBM NPs incubating with 4T1 breast cancer cells for 2, 4 and 8 hours measured by the flow cytometry.

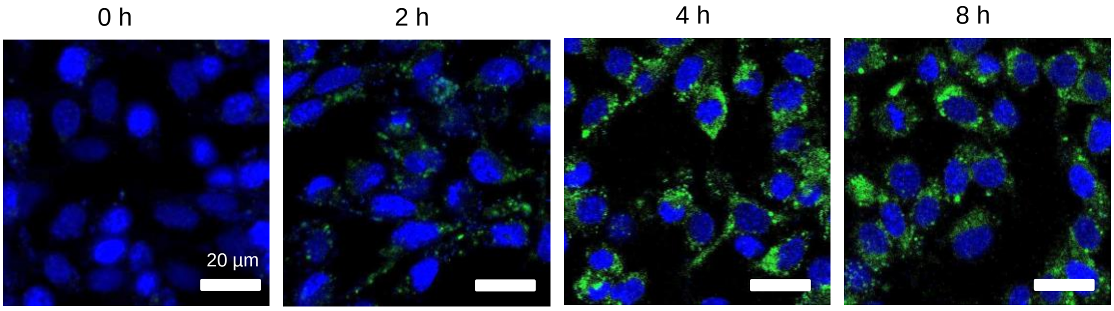

Fig. S12. CLSM images of 4T1 cells after treated with G_{FITC}TZ@Z-RBM for different time point.

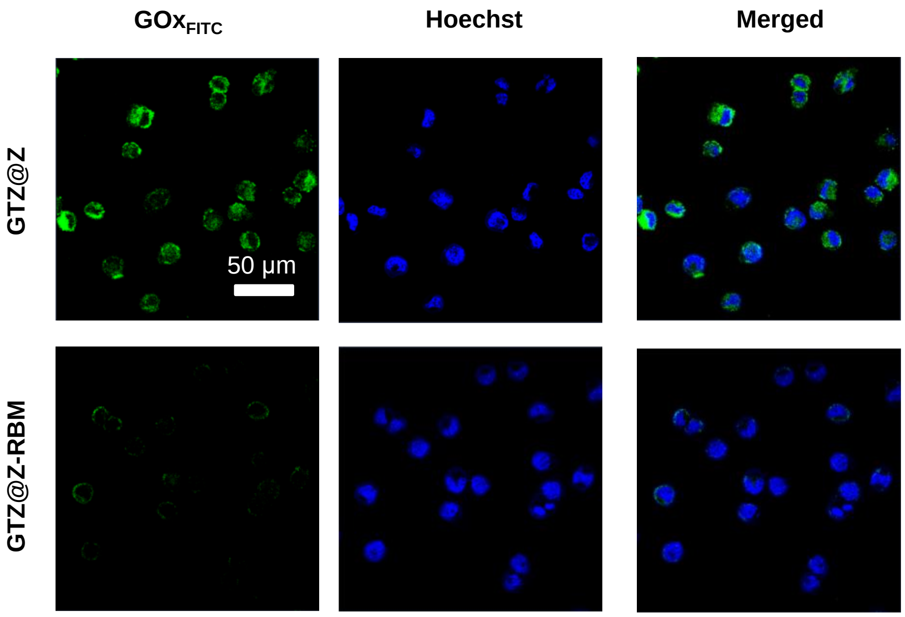

Fig. S13. Fluorescence micrographs of cellular uptakes by macrophages against GTZ@Z and GTZ@Z-RBM. The green fluorescence was associated with GOx_{FITC}, and blue fluorescence was expressed by Hoechst.

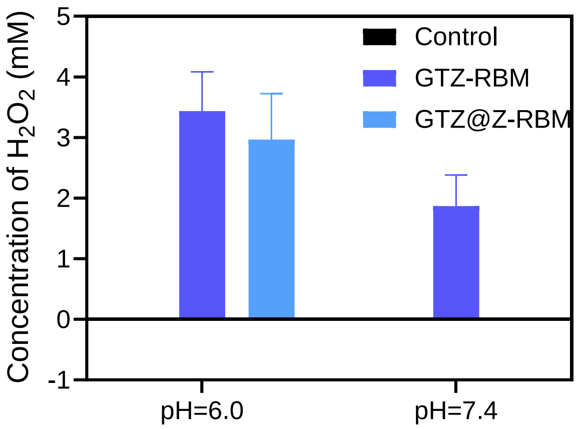

Fig. S14. The concentration of generated H_2O_2 in the cells after treated with GTZ-RBM and GTZ@Z-RBM under different pH (7.4 and 6.0).

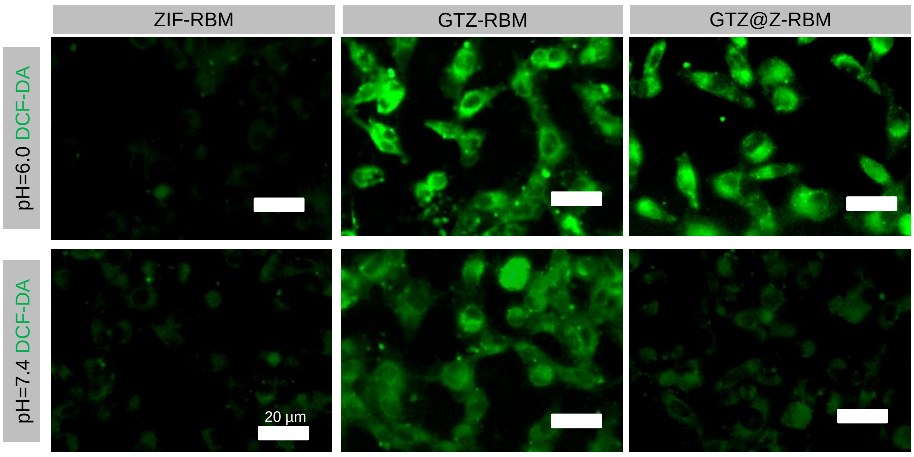

Fig. S15. The generation of ROS in the cells after treated with GTZ-RBM and GTZ@Z-RBM under different pH (7.4 and 6.0).

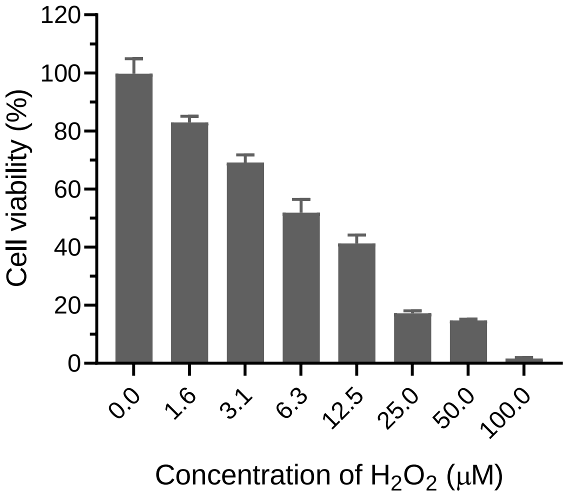

Fig. S16. Cell viabilities of 4T1 cells after treated with different concentration of H_2O_2.

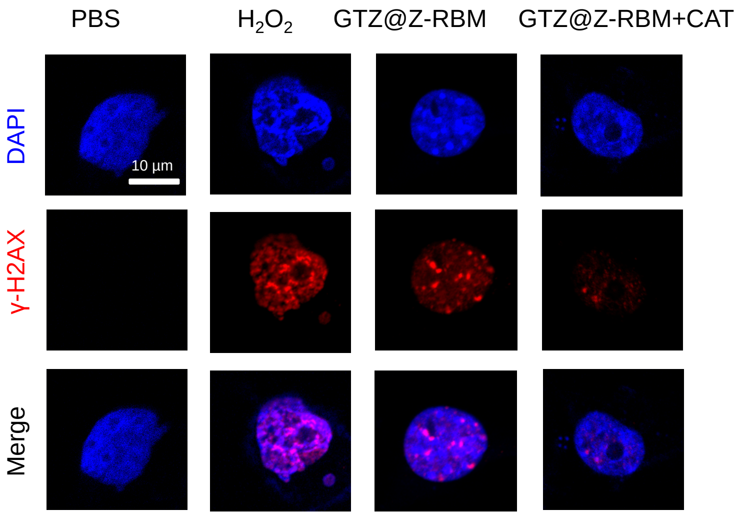

Fig. S17. Representative immunofluorescent images indicate the expression of

γ-H2AX foci in cell nuclei of 4T1 after different treatments under the pH 6.0, including PBS, H_2O_2 (1 mM), GTZ@Z-RBM (50 μg/mL), and GTZ@Z-RBM + catalase.

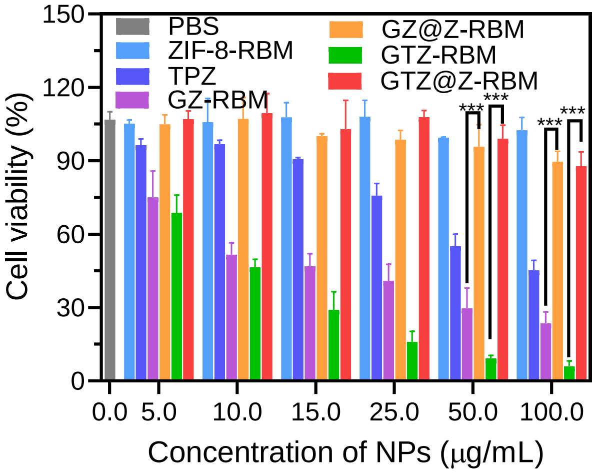

Fig. S18. Cell viabilities of 4T1 cells after treated with PBS, ZIF-8-RBM, TPZ, GZ-RBM, GZ@Z-RBM, GTZ-RBM, and GTZ@Z-RBM at the pH = 7.4.

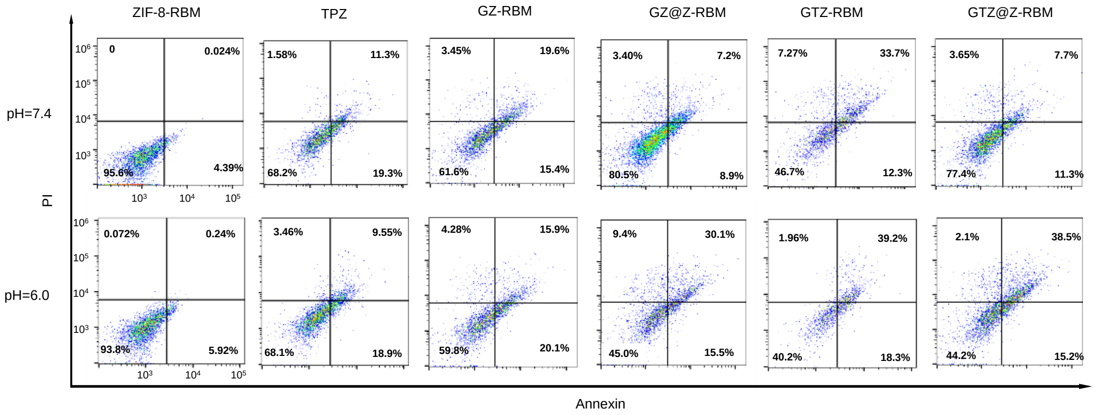

Fig. S19. Flow cytogram representing apoptosis assay based on Annexin V-FITC and propidium iodide staining of 4T1 cells after treated with ZIF-8-RBM, TPZ, GZ-RBM, GZ@Z-RBM, GTZ-RBM, and GTZ@Z-RBM under different pH for 4 h.

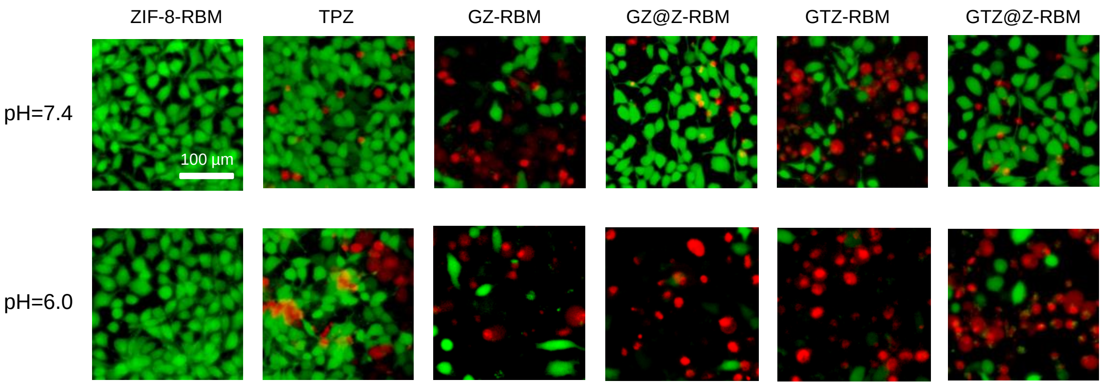

Fig. S20. Dead (red) and live (green) cells stained after treatments with ZIF-8-RBM, TPZ, GZ-RBM, GZ@Z-RBM, GTZ-RBM, and GTZ@Z-RBM under different pH for 4 h.

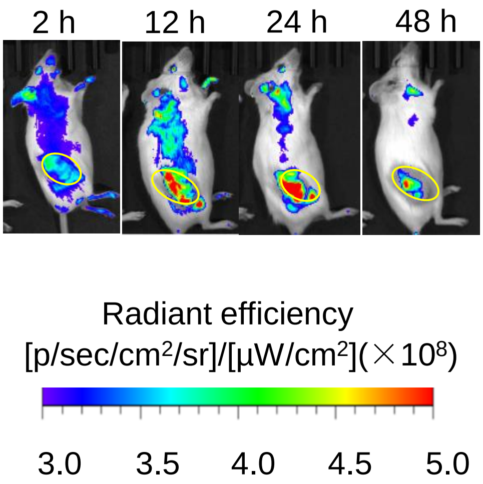

Fig. S21. Whole body fluorescence imaging of 4T1 tumor-bearing mice administrated with GTZ@Z-RBM-DiD. Yellow circles indicate the tumor regions.

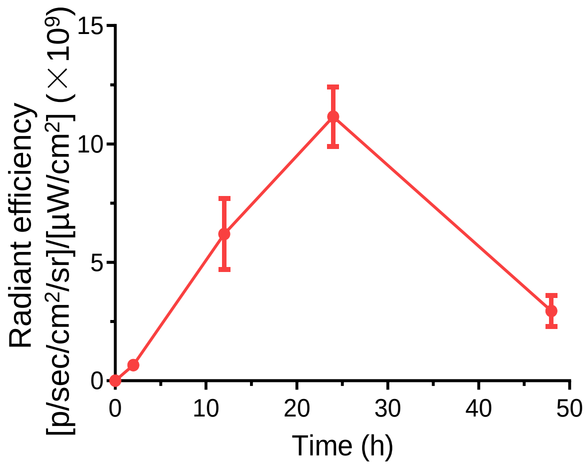

Fig. S22. DiD Fluorescence quantitative analysis at different time points post intravenous injection of DiD loaded GTZ@Z-RBM.

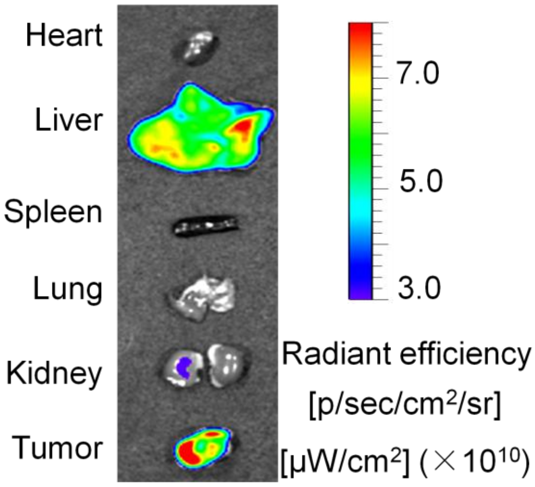

Fig. S23. Fluorescence imaging of major organs (heart, liver, spleen, lung, kidney) and tumor of the 4T1 tumor bearing mice. Images were taken at 48 h post injection of GTZ@Z-RBM.

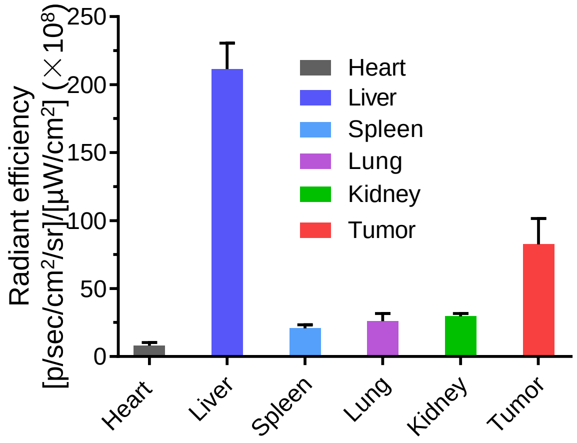

Fig.S24. Biodistribution of GTZ@Z-RBM labeled with DiD (% ID of gram of organ).

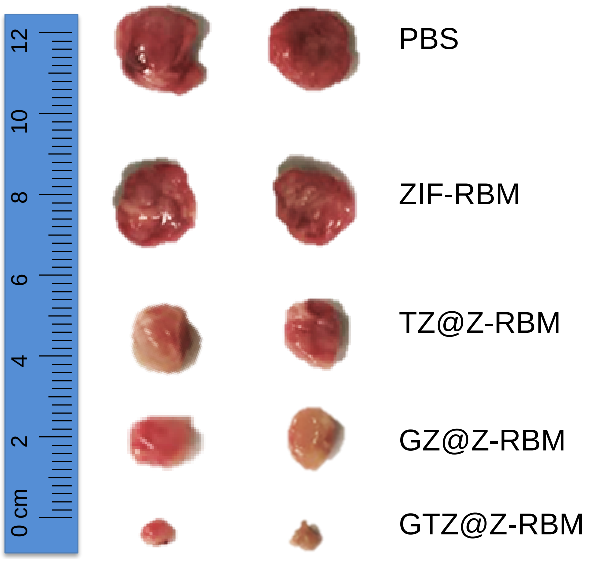

Fig. S25. Images of the tumor tissues obtained on day 18 post-treatment.

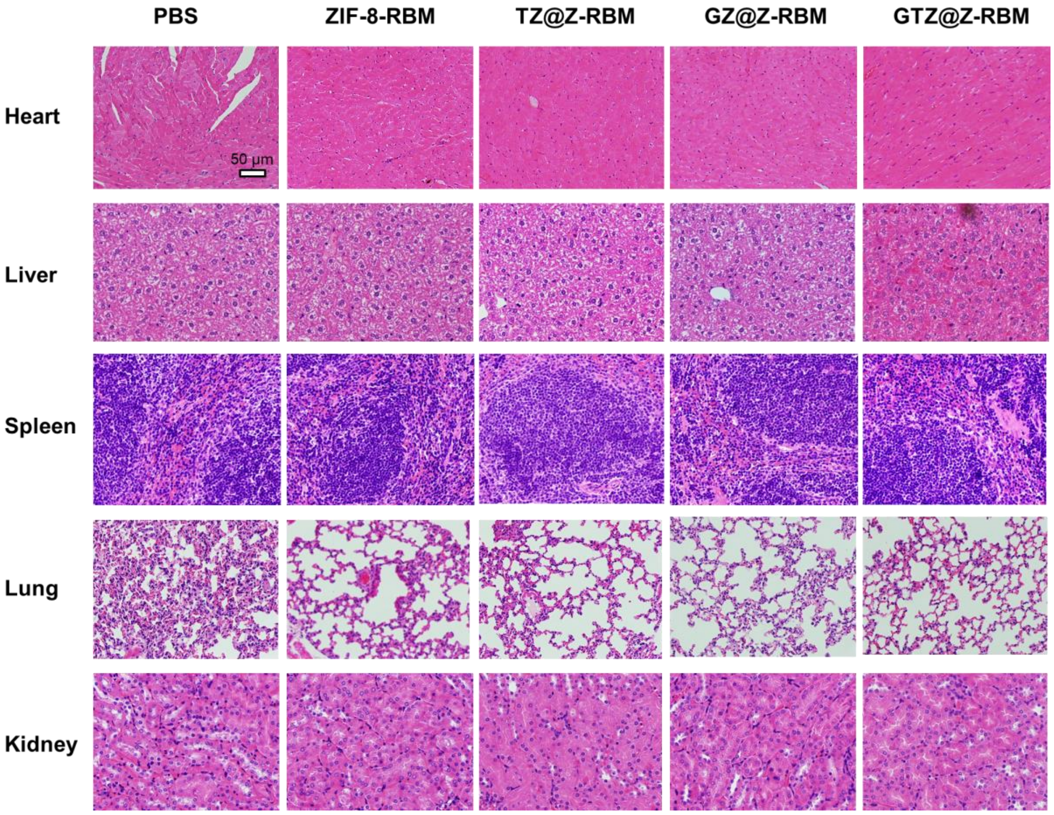

Fig. S26. Histological H&E-stained tissue sections of main tissues (heart, liver, spleen, kidney, and lung) from each group after various treatments on day 18.
